# Bridging the gap: the challenge of conducting clinical trials in sub-Saharan Africa

**DOI:** 10.1213/ANE.0000000000007461

**Published:** 2025-03-13

**Authors:** Arthur Kwizera, Laura A Hobbs, Daphne Kabatoro, Tom Bashford

**Affiliations:** 1 Department of Anaesthesia and Critical Care, Makerere University College of Health Sciences, Kampala, Uganda; 2 Association of Anesthesiologists of Uganda, Kampala, Uganda; 3 International Health Systems Group, Department of Engineering, University of Cambridge, UK; 4 NIHR Global Health Research Group on Acquired Brain and Spine Injury, University of Cambridge, Cambridge, UK; 5 East and North Hertfordshire NHS Trust, Stevenage, UK; 6 Cambridge Public Health Interdisciplinary Research Centre, University of Cambridge, Cambridge, UK; 7 Cambridge University Hospitals NHS Foundation Trust, Cambridge, UK

It is now almost ten years since the Lancet Commission on Global Surgery described the worldwide implications of a widespread lack of access to safe, affordable, surgical care, and the World Health Assembly adopted Resolution 68.15, calling for the strengthening of emergency and essential surgical and anaesthesia services.^1,2^ Although long-neglected by the international community, the World Health Organisation (WHO) now recognise that “surgery and anaesthesia care are essential to a comprehensive primary health care (PHC) approach and to a people-centred continuum of emergency, critical, and operative care (ECO) services.”^3^ In addition, the concept of National Surgical Obstetric and Anaesthesia Plans (NSOAPs), championed by WHA68.15, has been adopted by a number of countries to help integrate surgery and anaesthesia into their public health planning.^4^

Much of this focus has been on low- and middle-income countries, who house the majority of the global population and suffer the worst health outcomes from poor perioperative care provision. In sub-Saharan Africa (SSA) a huge range of initiatives to improve perioperative care have been undertaken in the past 20 years, from focussed training such as the SAFE courses pioneered by the UK and Ireland’s Association of Anaesthetists, to long-term capacity building through the development of postgraduate anaesthesia and critical care training.^5,6^ However, the perioperative research capacity of the continent has remained underdeveloped. The African Surgical Outcomes Study (ASOS) group have demonstrated the feasibility of large-scale high-quality studies by pan-African teams, but to date no comprehensive picture of perioperative and critical care research activity across SSA has been conducted.^7^

In this issue of *Anesthesia & Analgesia* Collier et al. from the University of Cape Town have sought to address this issue through a comprehensive scoping review of randomised controlled trials (RCTs) conducted in perioperative care across the continent.^8^ Their findings are sobering, but not unexpected, highlighting small sample sizes, poor outcome reporting, weak methodology and a limitation to a small number of countries and institutions. However, they should prompt a mature reflection on the role of RCTs in SSA, and the future research directions needed.

Clinical trials are fundamental to evidence-based treatment; nevertheless, executing them in low-resource environments, especially in Africa, poses distinct and intricate obstacles. Those working in anaesthesia and perioperative care on the continent must accommodate a lack of context-specific clinical evidence alongside the substantial burden of surgical and critical illnesses. Making pragmatic headway necessitates innovative solutions that harmonise local requirements, global standards, and equitable results. While Collier *et al* provide a useful synopsis that will undoubtedly inform policymakers, grant funders, and prospective collaborators as to the current trial landscape in Africa, we aim to describe some of the first-hand challenges, and opportunities, associated with conducting perioperative and critical care studies in Africa to contextualise their findings.

## Why conduct primary research in resource-poor settings?

Perhaps the first question that should be asked is why overburdened clinicians should consider conducting primary research, particularly randomised controlled trials, in the first place? Perioperative and critical care have seen enormous improvements in high resource settings over the past fifty years with a well-established pharmacopoeia and a suite of evidence-based interventions. Research in these areas is continuing, funded by large research grants and at the expense of wealthier nations. Improvement in SSA is likely to require complex interventions which may be less amenable to the constraints of RCT design, so why should SSA care providers be thinking about them at all? It might be argued that they have the ‘luxury’ of simply learning from the efforts of colleagues with the time and space to do research, occasionally combining these into small scale complex interventions or quality improvement efforts alongside their enormous burden of clinical care.

Against this position we would argue that our current evidence, from both basic science all the way through to clinical practice, is largely grounded in an unrepresentative global population centred on richer countries. As an example, research between colleagues in Cambridge and Kampala has demonstrated genetic difference in the KIR genes which drive pre-eclampsia in Ugandan as opposed to European populations, correlating with our clinical experience there are differences in their presentations.^9^ This then poses the question as to whether we should adopt clinical ‘evidence’ garnered in one population as applicable to another? We would argue the converse; that many of our suppositions about the existing evidence-base need to be questioned. In SSA we need confidence that the treatments we are using are the right ones for our patients, already some of the world’s most disadvantaged.

In addition, clinical research experience is a major component of training doctors of the future. Conducting high-quality RCTs is an excellent way of ensuring doctors in training understand the basic tenets behind the academic development of medicine, and paves the way for the development of larger trials or comparative effectiveness research. In SSA research activity remains, as in high-income countries, an area of personal interest associated with professional advancement and prestige. Failing to conduct high quality RCTs in SSA seems likely therefore to lead to poorer care, a frustrated workforce, and ultimately a migration of talented doctors away from the continent. However, we do see the need for SSA researchers to expand beyond the confines of basic science and trial research – the challenges of the continent mandate a revolution in how we think about clinical evidence and SSA researchers are positioned to be world leaders in comparative effectiveness research, qualitative research, complex interventions, and health systems science. However, as described by Conradie *et al* writing in the British Journal of Anaesthesia in 2018 in relation to the ASOS study, “Barriers to successful participation… related to resource limitations and not motivation of the clinician investigators.”^10^

## Infrastructure and Resource Constraints

One of the most significant challenges in African healthcare settings is inadequate infrastructure. This lived experience is reflected in several published studies, with many hospitals lacking the necessary resources for clinical care delivery.^11,12^ This has a concomitant impact on research ability with a failure in consistent supplies of electricity, laboratory capacity, and the digital tools necessary for data collection and management. Furthermore, there is a scarcity of trained research staff, as healthcare providers are often overstretched by routine clinical duties, leaving little bandwidth for research training. Outside of internationally funded studies, often led by the global north and with specific clinical interests, little to no research funding is available for clinicians to dedicate time to learning the skills necessary to conduct high-quality research which creates a high training burden for clinical trials to be conducted successfully. This barrier cannot be overstated, with many hospitals in SSA simply lacking the ’stuff, space, staff and systems’ required for high-quality research delivery.

## Ethical and Regulatory Complexities

Infrastructural gaps are compounded by ethical challenges and cultural factors, as illustrated by the Ugandan Children’s Oxygen Administration Strategies Trial (COAST).^13^ This aimed to evaluate oxygen therapy strategies for hypoxemic paediatric patients and was halted in 2019 due to ethical concerns about the risks to participants in a resource-constrained environment. Despite prior approval from ethics boards, questions arose about the study design and its appropriateness for a setting with limited oxygen infrastructure. Similarly, the Fluid Expansion as Supportive Therapy (FEAST) trial was halted early as the intervention arm, the supposedly higher standard of treatment, resulted in greater mortality.^14^ These studies underscore the need for both context-appropriate RCTs but also rigorous ethical oversight based in the communities being studied. It also highlights the need for ethical review boards to be adequately resourced to address the complexities of research in low-resource settings, ensuring participant protection and equitable distribution of rewards. However, maintaining ethical review boards is expensive, and there are complex priorities at play where funding from international studies is being made available to drive care improvements. Ethical review boards in resource-poor settings are often trying not simply to assess the ethical standards of the proposed research (which is in turn undermined where there is a deficit in time for research training of those running the boards), but also the complex political and personal agendas of those involved in the research. Furthermore, ingrained hierarchies and obtuse bureaucracies can frustrate clinical researchers. While many visiting researchers from the global north may feel that they bear the brunt of institutional bureaucracy in SSA, our experience is that it remains just as much a barrier to domestically-driven research efforts.

## Pragmatism and Adaptability in Trial Design: The ARISE Africa Trial

Given these constraints, there is a pressing need for pragmatic, adaptable, trial designs in diverse and resource-limited settings across SSA. One example is the recently completed Acute Respiratory Intervention StudiEs in Africa (ARISE-AFRICA) trial which was conducted across 17 largely rural hospitals in Uganda, investigating respiratory interventions while addressing real-time local constraints.^15^ Data capture was tailored to local conditions, allowing offline collection and subsequent integration into the trial database. Moreover, the trial engaged Uganda’s Ministry of Health, ensuring alignment with national priorities and facilitating the potential scalability of findings through policy.

The ARISE trial demonstrates how pragmatism and collaboration with local governments can enable successful trial execution without compromising scientific rigor. Regardless of the outcomes, its success emphasizes the value of designing trials that are not only robust but also context-sensitive, adaptable, and inclusive of local stakeholders. Notably, ARISE is the first RCT funded by the Wellcome Trust in SSA to be run entirely by an African research team, led by an anaesthesiologist.

## Leveraging Registry-Embedded Trials, Implementation Science and other approaches

Registry-embedded trials provide another innovative approach to overcome logistical and financial barriers. The Critical Care Asia-Africa Network leverages clinical registries to identify participants and collect real-world data for trials.^16^ These trials, embedded within routine care, are cost-effective and highly scalable. However, their success depends on the existence of reliable registries—an often-missing element in many African healthcare systems. The enrolment of country registries is ongoing, but a fundamental question arises as to how to incentivise local data collection in the face of heavy clinical duty and unreliable internet connectivity which creates delays in timely data collection on electronic platforms. Authorship positions on subsequent publications, a chance to develop research experience, and subsequent personal advancement go some way to encouraging hard-pressed clinicians to collect high-quality data – a need sometimes overlooked in registry efforts where those desiring the data are disconnected from the clinical reality of those tasked with collecting it.

Similarly, implementation science has significant potential in low-resource settings.^17^ By focusing on integrating evidence-based interventions into everyday practice, it addresses the "know-do gap." However, the approach may sacrifice some of the rigor associated with traditional randomized controlled trials (RCTs), and outcomes may be less generalizable across different contexts. Additionally, the question posed earlier remains: do ‘evidence-based’ interventions stand up to scrutiny across contexts? A range of other methodological approaches are being explored across a range of literatures including qualitative research, systems engineering, comparative effectiveness research, and a range of mixed-methods research modalities. These are likely to offer clinical researchers in SSA a diverse toolbox of research skills to address their unique challenges while contributing meaningfully to the international literature.^18^ It also provides areas of expertise in which they are well placed to become leaders on the global stage.

## Financial and Logistical Barriers

Insufficient funding for trials in Africa is a persistent challenge, often tied to donor-driven agendas that may not align with local health priorities – with perioperative and critical care persistently underfunded when compared to vertical health programmes which focus on specific conditions such as HIV or malaria. This can be frustrating where the rhetoric of funding agencies increasingly focusses on health systems strengthening – an area in which perioperative and critical care should be a central focus. Logistical challenges, such as transporting trial materials and maintaining participant follow-up in rural or remote areas, further inflate costs and complicate study execution. These barriers are particularly relevant in perioperative and critical care research, where the need for specialised equipment and consumables can be high.

## Sociocultural Challenges and Community Engagement

Community engagement is critical to the success of clinical trials, but in Africa is often hampered by mistrust of medical research and low health literacy. Effective engagement requires sustained efforts to build trust, culturally sensitive communication strategies, and a clear demonstration of the benefits of research for local populations. This can be a particular challenge where political, cultural, and religious dynamics are complicated by wealth imbalances and the structures by which healthcare is financed, making many groups deeply distrustful of the perceived medical elite. Finally, a particular challenge for perioperative and critical care physicians, is that they often do not feature in the public consciousness and do not have outpatient relationships with patients and their families which can help facilitate community engagement. Our experience of building community engagement in Kisoro, Uganda, for a community critical care project is that a deep local connection, local political engagement and endorsement, and constant dialogue can result in effective community engagement in critical care research in the most isolated communities, but that it is a sustained and complex effort by all parties.^19^

## Building Capacity and Sustainability

To address these challenges, there must be a concerted effort to build local capacity. Investments in infrastructure, the establishment of locally relevant registries, and training programs for African researchers are essential. Collaborative partnerships between African institutions and global research organizations can provide mentorship, funding, and technical expertise while ensuring that research aligns with local priorities. Crucially this requires a rebalancing of the global model of academic endeavour: co-authorship, co-ownership, and co-design must be central to all collaborative efforts, while locally owned SSA research must become the norm rather than the exception. Perioperative physicians also need to navigate entrenched local hierarchies to rise to positions of authority in their local institutions as well as Ministries of Health, in order to be able to direct research strategy and support perioperative and critical care research which has traditionally been under-represented and poorly advocated for. Uganda is an excellent example where this is changing, with Ministry buy-in as to the value of perioperative and critical care service, both in terms of clinical provision and research. The ASOS studies have been vital to building this awareness across the continent, and it now needs to be delivered at the national level.

## Conclusion

Conducting clinical trials in Africa is undeniably challenging, but the potential for transformative impact is immense. Innovations such as the ARISE Africa trial demonstrate that African research teams can deliver pragmatic studies tailored to local contexts which overcome infrastructural and ethical barriers while maintaining scientific rigor. Similarly, registry-embedded trials, qualitative research, systems engineering, and implementation science approaches hold promise for generating actionable evidence in resource-limited settings, albeit with their own limitations.

As a community, anaesthesiologists and perioperative care providers must advocate for sustainable investments in research capacity, infrastructure, and training while fostering ethical, collaborative partnerships. This advocacy needs to span all levels, from the World Health Organisation down to local institutions. By addressing these barriers, we can ensure that the benefits of clinical research extend to all populations, ultimately bridging the gap in global health equity.

## References

[1] Meara JG, Leather AJ, Hagander L, Alkire BC, Alonso N, Ameh EA, Bickler SW, Conteh L, Dare AJ, Davies J, Mérisier ED (2015). Global Surgery 2030: evidence and solutions for achieving health, welfare, and economic development. Lancet.

[2] Strengthening emergency and essential surgical care and anaesthesia as a component of universal health coverage.

[3] Surgical and anaesthesia care.

[4] Peck GL, Hanna JS (2018). The National Surgical, Obstetric, and Anesthesia Plan (NSOAP): Recognition and Definition of an Empirically Evolving Global Surgery Systems Science Comment on "Global Surgery - Informing National Strategies for Scaling Up Surgery in Sub-Saharan Africa". Int J Health Policy Manag.

[5] Boyd N, Sharkey E, Nabukenya M, Tumukunde J, Sipuka N, Zyambo M, Walker I, Hart J, Byrne-Davis L (2019). The Safer Anaesthesia from Education (SAFE)®paediatric anaesthesia course: educational impact in five countries in East and Central Africa. Anaesthesia.

[6] Lipnick M, Mijumbi C, Dubowitz G, Kaggwa S, Goetz L, Mabweijano J, Jayaraman S, Kwizera A, Tindimwebwa J, Ozgediz D (2013). Surgery and anesthesia capacity-building in resource-poor settings: description of an ongoing academic partnership in Uganda. World J Surg.

[7] Biccard BM, Madiba TE, Kluyts HL, Munlemvo DM, Madzimbamuto FD, Basenero A, Gordon CS, Youssouf C, Rakotoarison SR, Gobin V, Samateh AL (2018). Perioperative patient outcomes in the African Surgical Outcomes Study: a 7-day prospective observational cohort study. Lancet.

[8] Collier L, Hohlfeld ASJ, Biccard BM Mapping Perioperative Care Randomized-Controlled Trials in sub-Saharan Africa: A Scoping Review [REF as per journal].

[9] Nakimuli A, Chazara O, Hiby SE, Farrell L, Tukwasibwe S, Jayaraman J, Traherne JA, Trowsdale J, Colucci F, Lougee E, Vaughan RW (2015). A KIR B centromeric region present in Africans but not Europeans protects pregnant women from pre-eclampsia. Proc Natl Acad Sci U S A.

[10] Conradie A, Duys R, Forget P, Biccard BM (2018). Barriers to clinical research in Africa: a quantitative and qualitative survey of clinical researchers in 27 African countries. Br J Anaesth.

[11] Hodges SC, Mijumbi C, Okello M, McCormick BA, Walker IA, Wilson IH (2007). Anaesthesia services in developing countries: defining the problems. Anaesthesia.

[12] Bashford T (2014). Anaesthesia in Ethiopia: providers' perspectives on the current state of the service. Trop Doct.

[13] Maitland K, Kiguli S, Opoka RO, Olupot-Olupot P, Engoru C, Njuguna P, Bandika V, Mpoya A, Bush A, Williams TN, Grieve R (2018). Children's Oxygen Administration Strategies Trial (COAST): A randomised controlled trial of high flow versus oxygen versus control in African children with severe pneumonia. Wellcome Open Res.

[14] Maitland K, Kiguli S, Opoka RO, Engoru C, Olupot-Olupot P, Akech SO, Nyeko R, Mtove G, Reyburn H, Lang T, Brent B (2011). Mortality after fluid bolus in African children with severe infection. N Engl J Med.

[15] Kwizera A, Kabatoro D, Owachi D, Kansiime J, Kateregga G, Nanyunja D, Sendagire C, Nyakato D, Olaro C, Audureau E, Mekontso Dessap A (2024). Respiratory support with standard low-flow oxygen therapy, high-flow oxygen therapy or continuous positive airway pressure in adults with acute hypoxaemic respiratory failure in a resource-limited setting: protocol for a randomised, open-label, clinical trial - the Acute Respiratory Intervention StudiEs in Africa (ARISE-AFRICA) study. BMJ Open.

[16] CRIT CARE ASIA (2020). Establishing a critical care network in Asia to improve care for critically ill patients in low- and middle-income countries. Crit Care.

[17] Takoutsing BD, Ikwuegbuenyi C, Umutoni A, Ogunfolaji O, Bankole NDA, Mpokota R, Adegboyega G, Kanmounye US, Dokponou YCH (2021). Health systems strengthening, dissemination, and implementation science in Africa: *quo vadis*?. Pan Afr Med J.

[18] Patel J, Tolppa T, Biccard BM, Fazzini B, Haniffa R, Marletta D, Moonesinghe R, Pearse R, Vengadasalam S, Stephens TJ, Vindrola-Padros C (2022). Perioperative Care Pathways in Low- and Lower-Middle-Income Countries: Systematic Review and Narrative Synthesis. World J Surg.

[19] Kisoro District ‘ICU’ game changer in Uganda’s Critical Care Medicine.

